# Evaluation of Intraventricular/Intrathecal Antimicrobial Therapy in the Treatment of Nosocomial Meningitis Caused by Multidrug-Resistant Gram-Negative Bacteria after Central Nervous System Surgery

**DOI:** 10.1155/2021/9923015

**Published:** 2021-08-27

**Authors:** Nagehan Didem Sari, Sevim Baltali, Istemi Serin, Veysel Antar

**Affiliations:** ^1^University of Health Science, Istanbul Training and Research Hospital, Department of Infectious Diseases and Clinical Microbiology, Istanbul, Turkey; ^2^University of Health Science, Istanbul Training and Research Hospital, Department of Anesthesiology and Reanimation, Istanbul, Turkey; ^3^University of Health Science, Istanbul Training and Research Hospital, Department of Hematology, Istanbul, Turkey; ^4^University of Health Science, Istanbul Training and Research Hospital, Department of Neurological Surgery, Istanbul, Turkey

## Abstract

**Introduction:**

Postoperative meningitis (POM) is an infection with high mortality and morbidity following central nervous system surgery due to trauma or tumor. Intrathecal/intraventricular (IT/IVT) antibiotic administrations have been considered as the last treatment options for multidrug-resistance (MDR) Gram-negative bacteria that do not respond to intravenous (IV) regimens. IT/IVT can bypass the blood-brain barrier, obtain a more effective antibiotic concentration in CSF, and reduce systemic side effects. We aimed to determine the characteristics of postoperative patients who were diagnosed with MDR POM during follow-up in our intensive care unit (ICU). *Material and Methods*. In this study, POM patients who were followed up in ICU after the central nervous system intervention between January 2016 and December 2019 and whose MDR Gram-negative bacteria were isolated from CSF were evaluated. As soon as the patients were diagnosed with POM, a catheter was inserted and treatment was started.

**Results:**

Microbiological eradication was achieved in 3 ± 0.8 days with 30 mg/day amikacin treatment in POM due to *K. pneumoniae* and 3.7 ± 1.95 days with colistin sodium 10 mg/day treatment in POM due to *A. baumannii* via IT/IVT catheter. IT/IVT treatment was utilized for a median of 10 days and continued until the defined cure criteria were achieved. While cure was achieved in 6 of 14 POM cases, 8 of them were exitus. *Discussion and Conclusion*. To avoid the severe consequences of postoperative meningitis, acting fast and adding IT/IVT methods to parenteral administration routes by considering the distribution of MDR microorganisms within the hospital while planning effective treatment will increase the clinical success.

## 1. Introduction

Postoperative meningitis (POM) is an infection with high mortality and morbidity following central nervous system surgery due to trauma or tumor with an incidence of 0.8%–1.8% [1–3]. It is mainly caused by cutaneous microorganisms such as coagulase-negative staphylococci, *Staphylococc*us aureus (*S.* aureus), and Propionibacterium acnes (P. acnes) [4]. Gram-negative bacteria also play a role. Antibiotic prophylaxis is recommended for neurosurgery which is mainly directed against Gram-positive bacteria. Nosocomial staphylococci usually carry methicillin resistance. Current guidelines recommend treatment with either an antipseudomonal cephalosporin or an antipseudomonal carbapenem to cover aerobic Gram-negative bacteria and vancomycin to cover staphylococci and *P. acnes* [4]. The choice between a cephalosporin and a carbapenem for empirical therapy should be based on the increasing prevalence of broad-spectrum *β*-lactamase-producing bacteria. With high prevalence of nosocomial infections caused by carbapenem-resistant *Acinetobacter baumannii (A. baumannii)* and other Gram-negative bacteria, colistin should be considered in combination with carbapenems until drug sensitivities are determined. The use of colistin alone is not recommended due to its low intrinsic clinical efficacy and poor CSF penetration [4].

Due to the increased drug resistance, the use of different groups of antibiotics, which cannot cross the blood-brain barrier, with different administration routes has become a current issue [5]. Intrathecal/intraventricular (IT/IVT) antibiotic applications come into prominence especially in the treatment of meningitis caused by multidrug-resistant (MDR) Gram-negative bacteria developed after surgical interventions [6–9].

The rate of mortality is higher in patients with Gram-negative bacterial intracranial infections and MDR Gram-negative agents have also more complicated treatment process. *A. baumannii, Klebsiella pneumoniae (K. pneumoniae)*, and *Pseudomonas aeruginosa (P. aeruginosa)* are the most common MDR Gram-negative agents [10, 11]. Against MDR or extensively drug-resistant (XDR) Gram-negative bacteria, there are only a few drugs such as polymyxins and aminoglycosides; however, due to low brain penetration, intracranial infection does not show any improvement even when treated with these antimicrobial agents. Thus, IT or IVT antibiotic administrations have been considered as the last treatment options for MDR/XDR Gram-negative bacteria that do not respond to intravenous (IV) regimens [12]. IT/IVT can bypass the blood-brain barrier, obtain a more effective antibiotic concentration in CSF, and reduce systemic side effects [12]. Potential neurotoxicity such as chemical meningitis and ventriculitis cannot be ignored, but these reactions have been reported to be mild and also reversible [10, 12]. The most commonly used IT/IVT agents are aminoglycosides (gentamycin, tobramycin, netilmicin, amikacin, and streptomycin), polymyxins (colistin and polymyxin B), daptomycin, glycopeptides (vancomycin and teicoplanin), tigecycline, and antifungal agents such as amphotericin B and caspofungin [12].

In this article, we aimed to determine the demographic characteristics, laboratory values, and empirical and antimicrobial treatment options in postoperative patients who were diagnosed with MDR POM during follow-up in our intensive care unit (ICU).

## 2. Material and Methods

In this retrospective single-center study, cases of POM who were followed up in Istanbul Training and Research Hospital Intensive Care Unit after the central nervous system intervention between January 2016 and December 2019 and whose MDR Gram-negative bacteria were isolated from CSF were evaluated.

MDR definition was made in accordance with Magiorakos et al. [13]. All patients were administered ceftriaxone or cefazolin during the induction of anesthesia. The definition of nosocomial meningitis/ventriculitis (NM) was based on the classification of the American Centers for Disease Control and Prevention (CDC) [14]. According to the CDC, the case definition is as follows:

1. >38°C fever, detected 72 hours after the operation
2. Agent isolation from the CSF
3. Leukocyte count >10/mm³, protein >45 mg/dL, and glucose <40 mg/dL in CSF
4. No reproduction in other cultures taken simultaneously with CSF (such as blood, tracheal aspirate, and urine)

Meningitis with another agent was accepted as a new case 7 days after the eradication of the first agent from CSF. After 30 days of eradication of agent, sterilization of CSF and recovery without sequelae were considered as a cure.

Demographic, clinical, and treatment applications of the cases meeting our criteria were obtained from computer and electronic file records. Causes of intervention, administered antibiotics, microorganisms isolated from CSF and their sensitivities, treatment methods, and disease outcomes were all recorded. Response to treatment was evaluated with daily patient visits, clinical course, and biochemical and microbiological examinations of CSF samples taken every 48 hours.

CSF samples for POM diagnosis were taken from lumbar puncture and ventricular or lumbar drainage catheter. CSF and blood culture were taken simultaneously and kept at room temperature while being delivered to the laboratory. All CSF samples were sown in 5% sheep blood agar and chocolate agar (containing X and V content) and incubated at 37°C for 72 hours in 5% CO_2_ medium. The BACTEC™ Automated Blood Culture System (Becton Dickinson) was used for blood cultures.

The standard microbiological evaluation was made on the isolates (Gram staining, colony morphology, catalase, oxidase, and coagulase tests). VITEK 2 system (bioMérieux, France) was used for bacterial identification and susceptibility.

### 2.1. Administration of IT/IVT Agents

As soon as the patients were diagnosed with POM, a catheter was inserted and treatment was started. Due to low CSF penetration of IV drugs and nosocomial resistant microorganisms, simultaneous IT/IVT treatment process was aimed. Antibiotherapy was revised according to culture results and antibiograms.

The IT/IVT agents amikacin and colistin were administered as described below:

1. Amikacin 30 mg/day and colistin 10 mg/day were administered
2. The administration was made through the intraventricular/lumbar catheter; firstly, 5 cc of CSF was sampled and the catheter line was emptied
3. All antibiotics were diluted with 5 cc 0.9% NaCl and given through the catheter and the drainage was closed for 1 hour
4. CSF culture was taken every 48 hours
5. Sterility of the CSF samples taken 3 times in a row was considered as cure

### 2.2. Statistics

Results are presented as mean ± standard deviation and median (min–max) for continuous variables and n (percentage) for categorical variables.

Ethics committee approval was received from Istanbul Training and Research Hospital Clinical Research Ethics Committee on 09/08/2019 (decision no. 1940).

## 3. Results

A total of 1562 neurosurgical operations were performed in Istanbul Training and Research Hospital between January 2016 and December 2019 with different indications. Postoperatively, 1023 of these cases were followed up in the ICU.

**Figure 1:**
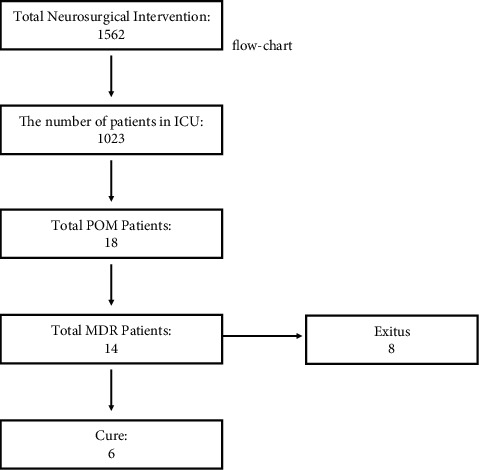
Patient flowchart.

POM was detected in 18 of 1023 patients with ICU follow-up (1.75%). Fourteen of 18 detected cases were diagnosed with MDR POM (77.7%) (Figure 1). 71.42% (10/14) of the cases were male and the mean age was 48.28 ± 16.93 years (range: 16–75 years). Operation indications were intracranial bleeding (8 cases), hydrocephalus (2 cases), traumatic brain injury (2 cases), intraventricular bleeding (1 case), and ventriculoperitoneal shunt revision (1 case). Extraventricular drainage (EVD) catheter (10 cases) and lumbar drainage catheter (4 cases) were placed to reduce intracranial pressure in patients. The prevalence of MDR POM was 0.9% in total.

In patients with MDR, *A. baumannii*, 71.43% (10/14), and *K. pneumoniae*, 28.57%, were the causative agents. The average time until POM was detected as 12.07 ± 11.84 days. Vancomycin (14 cases), meropenem (7 cases), ceftazidime (6 cases), and ceftriaxone (1 case) were used as empirical treatments. Before IT/IVT therapy, empirical antibiotics were applied for 10 ± 8.8 days.

At the time of diagnosis, CSF glucose was 11.93 ± 11.83 g/dL, protein was 336.69 ± 219.89 mg/dL, and leukocyte count was 886.43 ± 364.81/mm³. Meropenem-colistin combination was used IV in 7 of 10 patients diagnosed with *A. baumannii*, with tigecycline-colistin in 2 patients and tigecycline-colistin-vancomycin in 1 patient. While meropenem-amikacin combination was used in 3 out of 4 patients diagnosed with *K.* pneumoniae, meropenem-vancomycin was used in 1 patient. Microbiological eradication was achieved in 3 ± 0.8 days with 30 mg/day amikacin treatment in POM due to K. pneumoniae and 3.7 ± 1.95 days with colistin sodium 10 mg/day treatment in POM due to *A. baumannii* via IT/IVT catheter. IT/IVT treatment was utilized for a median of 10 days and continued until the defined cure criteria were achieved. There was no complication observed related to the administration of IT/IVT amikacin and colistin.

While cure was achieved in 6 of 14 POM cases, 8 of them were exitus. Of the 6 patients who were cured, 3 died due to cerebral hemorrhage; the other 3 patients were discharged with permanent shunt. Demographic data, operation indications, prediagnosis CSF findings, and CSF findings during the period when microbiological eradication was achieved, as well as empirical and antibiotic treatments against the agent, are detailed in Table 1.

## 4. Discussion

The exact diagnosis of POM is made with a combination of clinical signs plus microbiological and biochemical analysis of the CSF. Gram-negative bacteria are responsible for 60–70% of POMs. *A. baumannii* has an important place among Gram-negative bacteria. Long-term use of broad-spectrum and inappropriate antibiotics, use of ventricular catheters for more than 5 days, and ventriculostomy are risk factors for *A. baumannii* meningitis [15–22]. While the mortality rate in meningitis caused by *A. baumannii* varies between 30 and 50%, this rate is reported to be as high as 50–70% in MDR A. *baumannii* meningitis [23]. In our study, prevalence of POM related to *A. baumannii* was 71.43% and 60% of our cases lost their lives.

In a study conducted in Turkey in 2020, Gram-negative meningitis cases treated with IT/IVT were examined [24]. The most common microorganism in 13 patients included in the study was *A. baumannii* (8/13). Colistin was used consecutively in 8 patients, amikacin in 4 patients, and amikacin plus colistin in 1 patient. The 28-day infection-related mortality was 15% with 2 patients. In our study, mortality was 57.1% with 8 patients in a total of 14 MDR POM cases. In patients with MDR, *A. baumannii* in 71.43% (10/14) and *K. pneumoniae* in 28.57% of patients were the causative agents. The most commonly used antimicrobial agent was colistin.

Another study in 2019 [25] examined a total of 25 POM cases with XDR *A. baumannii* infection. All patients received IVT colistin and the mean duration of colistin usage was 13.4 ± 2.8 days. The time to obtain a negative CSF culture was 8.9 ± 4.0 days. In this study, mortality was 20% with 5 patients. In another study from 2018 [26], a retrospective cohort was conducted in patients who were diagnosed with POM and admitted to ICU receiving IVT antibiotic therapy. All 105 patients enrolled in the study received systemic antimicrobial therapy in combination with at least one dose of an IVT antimicrobial agent. IVT vancomycin was used in 52.4% of the patients. Overall mortality was 18.1% with 19 patients. CSF culture sterilization was achieved in 88.4% of patients. In another study in 2018 [27], a total of 61 cases of POM infected with MDR microbial agents were examined. The rate of MDR *A. baumannii* infection was 33.64%. The patients were treated with polymyxin B. Compared to the IV treatment group, the IT/IVT treatment group had lower 28-day mortality (55.26% and 8.70%, *p*=0.01) and higher rates of clinical and antimicrobiological efficacy (95.65% versus 23.68%, *p* < 0.001%; 91.30% versus 18.42%, *p* < 0.001), respectively. In our study, microbiological eradication was achieved in 3 ± 0.8 days with 30 mg/day amikacin treatment in POM due to *K. pneumoniae* and 3.7 ± 1.95 days with colistin 10 mg/day treatment in POM due to A. baumannii via IT/IVT catheter. Cure was achieved in 6 of 14 POM cases and 8 of them were exitus.

The fact that A. baumannii is increasingly resistant to many antibiotics including carbapenem group is an important problem in treatment in healthcare-related infections [28]. Tuon et al. detected carbapenem resistance in 40.9% of nosocomial meningitis. In addition, they identified the most important cause of mortality as the inappropriate administration of antibiotic therapy within five days of receiving CSF culture [18]. In our study, all *A. baumannii* strains were resistant to carbapenem and the empirical treatments applied were insufficient.

At this point, it would be good to mention ceftazidime/avibactam. Both the coverage of carbapenem-resistant Gram-negative bacteria and the better CSF penetration compared to other beta-lactam antibiotics cause significant advantages [11]; however, since it is not in use in our country, it is not included in our study.

As a result of blood-brain barrier impairment and alteration of drug pharmacokinetics due to CNS infections, intravenously administered drugs could not reach the desired effective concentrations. IT/IVT administration is generally preferred in the presence of resistant microorganisms, where systemic IV antibiotic therapy is thought to be inadequate. The main reason why these methods are not preferred in the first place are dose-dependent toxic reactions such as seizure, chemical ventriculitis, and transient hearing loss (after gentamicin and vancomycin) shown in previous studies [29–31]. On the other hand, there are some studies reporting that the side effects related to polymyxin B, colistimethate sodium, and vancomycin are negligible and that these minimal side effects are also dose-dependent [32–34].

Rodriquez et al. compared different routes of antibiotic administration in nosocomial *A. baumannii* infection and reported that both intraventricular and intrathecal colistin treatments are useful and safe options [22]. It was reported that colistin, gentamicin, amikacin, and polymyxin B antibiotics can be used in the treatment of resistant *A. baumannii* strains via IT/IVT [35]. In a meta-analysis in 2018 [36], 11 studies with 348 patients met eligibility criteria. In this study comparing IVT plus IV treatment and IV treatment, IVT plus IV treatment was statistically superior to IV therapy alone in eradication and mortality.

Remes et al. firstly reported that, alternatively, meropenem and netilmicin therapy will also be effective without observing side effects [33]. In a study of 21 patients, Khan et al. reported that IT/IVT antibiotic administration is beneficial and has minimal side effects in cases of postoperative Gram-negative meningitis and ventriculitis that do not respond to standard therapy [34]. In our retrospective study, no complication was observed with the administration of IT/IVT amikacin and colistin.

In our small series study, doses of the administered drug(s) and clinical data of the patients could not be compared with detailed statistical methods, as the retrospective study was insufficient in the clinical determination of drug side effects in the cases that were followed up under sedation in the intensive care unit.

## 5. Conclusion

As a result, we believe that, in selected cases, to avoid the severe consequences of postoperative meningitis, acting fast and adding IT/IVT methods to parenteral administration routes by considering the distribution of MDR microorganisms within the hospital while planning effective treatment will increase the clinical success.

## Figures and Tables

**Table 1:** Demographic data, operation indications, microbiological agent, empirical and agent targeted antibiotic preferences, and durations of therapies.

Case	Age/gender	Operation indication	Days of POM development	Empirical therapy	Microbiological agent	Agent targeted therapy (IV)	Duration (days)	IV/IVT therapy	Duration (days)	Last status
1	52/M	Intraventricular hemorrhage	12	Mem, Va	*A. baumanni*	Mem, Col	5	Col	5	Exitus
2	56/M	Hydrocephalus	1	Caz, Va	*A. baumanni*	Tig, Col	21	Col	10	Cure
3	54/M	Aneurysm	11	Caz, Va	*A. baumanni*	Mem, Col	5	Col	5	Exitus
4	54/M	Hydrocephalus	34	Caz, Va	*K. pneumoniae*	Mem, Ak	15	Ak	10	Cure
5	39/F	Aneurysm	9	Mem, Va	*A. baumanni*	Mem, Col	7	Col	7	Exitus
6	42/M	Aneurysm	15	Caz, Va	*A. baumanni*	Mem, Col	12	Col	7	Exitus
7	16/M	Aneurysm	7	Mem, Va	*A. baumanni*	Tig, Col	20	Col	14	Cure
8	49/M	Aneurysm	10	Caz, Va	*A. baumanni*	Tig, Va, Col	20	Col	14	Cure
9	71/M	Aneurysm	42	Caz, Va	*A. baumanni*	Mem, Col	14	Col	10	Cure
10	16/M	Shunt revision	2	Mem, Va	*K. pneumoniae*	Mem, Ak	13	Ak	10	Exitus
11	42/F	Trauma	6	Cro, Va	*A. baumanni*	Mem, Col	12	Col	10	Exitus
12	52/M	Aneurysm	12	Mem, Va	*A. baumanni*	Mem, Col	10	Col	10	Exitus
13	75/M	Trauma	2	Mem, Va	*K. pneumoniae*	Mem, Va	21	Ak	10	Cure
14	58/F	Aneurysm	6	Mem, Va	*K. pneumoniae*	Mem, Ak	7	Ak	7	Exitus

IV: intravenous; IVT: intraventricular; Mem: meropenem; Va: vancomycin; Caz: ceftazidime; Cro: ceftriaxone; Tig: tigecycline; Col: colistimethate sodium; Ak: amikacin.

## Data Availability

The clinical data used to support the findings of this study are available from the corresponding author upon request.

## Abbreviations

POM: Postoperative meningitis
*S. aureus*: *Staphylococcus aureus*
*P. acnes*: *Propionibacterium acnes*
*K. pneumoniae*: *Klebsiella pneumoniae*
*P. aeruginosa*: *Pseudomonas aeruginosa*
XDR: Extensively drug-resistant (XDR)
MDR: Multidrug-resistant
ICU: Intensive care unit
CSF: Cerebrospinal fluid
NM: Nosocomial meningitis
CDC: American Centers for Disease Control and Prevention
EVD: Extraventricular drainage
IVT: Intraventricular
IT: Intrathecal.
